# 

**Published:** 1911-02-18

**Authors:** 


					By Sir WILLIAM BENNETT, K.C.V.O., F.R.C.S.
RECENTLY PUBLISHED. Crown 8vo. 58. net.
INJURIES AND DISEASES OF
THE KNEE JOINT.
With 34 Illustrations.
IT I S 33 33 T <5c CO.
Also, 8vo. 6s. FOURTH EDITION.
MASSAGE AND MOVEMENTS IN
RECENT FRACTURES;
Sprains and their Consequences; Rigidity of
the Spine and the Management of Stiff Joints.
With 23 Illustrations.
LONG-MANS, G-BEEN <5c CO.
Just Issued. Ninth Edition, Rewritten and Enlarged, 9s. net.
PHARMACY, MATERIA MEDICA & THERAPEUTICS.
Being a Commentary on the B.P. and its Drugs, with their Preparations,
and on all the new Non-official Remedies in use, with Chapters on the
different processes used in Dispensing, Prescription-writing, Latin
Glossary, &c. By Sip W. WHITLA, M.D., LL.D., Professor, Materia
Medica, Queen's University, Belfast, and Senior Physician, Royal Victoria
Hospital.
By the same Author. New Work. 1,900 pages. Grown 8vo. 25s. net.
PRACTICE OF MEDICINE.
Containing in two Handy Volumes a complete and independent Article on
the Etiology, Symptoms, and Diagnosis of every Medical Disease; arranged
in Dictionary Form for Practitioners and Students.
London: Bailli&re, Tindall & Cox, 8 Henrietta Street, W.O.
FIFTH EDITION. With 50 Illustrations. 5/- net.
THE SCHOTT METHODS OF THE TREATMENT
OF CHRONIC DISEASES OF THE HEART.
With an Account of the Nauheim Baths and of their Therapeutic Exercises
By W. BEZLY THORNE, M.D., M.R.C.P.
London: J. & A. Churchill, 7 Great Marlborough Street.
By A. H. TUBBY, M.S.
DEFORMITIES.
Six hundred pages, 300 Illustrations. Price 17s.
" Standard work on the subject in the English language."
British Medical Journal.
SURGERY OF PARALYSES.
By A. H. TUBBY, M.S.Lond., F.R.C.S.Eng., and
ROBERT JONES, F.R.C.S.E.
Illustrated by 93 figures and 68 cases. Pp. 301. 10s. net.
" Can confidently recommend it as a practical summary of the most
modern -views."?Edinburgh Medical Journal.
" Indicates original and successful methods of treatment."
American Journal of Orthopaedic Surgery.
London : Macmillan & Co., Ltd. New York : The Macmillan Oo.
A NEW BOOK. Crown 8vo. 5s. net.
THE NEWER REMEDIES (a Practical Guide To).
By J. M. Fortescue-Brickdale, M.A., M.D. Oxon., Phys., Olifton
Coll.; Asst. Phys., B'tol Roy. Infy.; Lect. Pharm., Univ. of Oxford ; Clin.
Lect., Univ. of Bristol. An account of the Principal New Drugs, with
indications as to their value and employment.
" Will help the harassed practitioner."?Hospital.
" We very cordially recommend it."?South African Medical Record.
Bristol: J. Wright & Sons, Ltd. London : Simpkin & Co., Ltd.
THERAPEUTICS OF THE CIRCULATION
By Sir LAUDER BRUNTON, Bart., M.D., D.Sc., F.R.C.P., &c.,
Consulting Physician to St. Bartholomew's Hospital.
Published under thk Auspices op the University of London.
With numerous Diagrams. Medium 8vo. 7s. 6d. net.
JOHN MURRAY, Albemarle Street, W.
" The Prescriber.
99
A Monthly Journal dealing with Therapeutics, Pharma-
cology, and the Newer Remedies.
" One of the most useful publications the practitioner can subscribe
to."?See The Hospital, Jan. 7,1911.
Subscriptions for 1911 should be sent NOW. .
Five Shillings per annum, post free anywhere. Specimen uopy ?
"THE PRESCRIBER" Offices, 137 George St., EDINBURGH*
February 18, 1911 THE HOSPITAL
it)
Cassell's Medical Works.
READY MARCH lOtln.
New Edition, Revised Throughout. 686 pages demy 8vo., 21s. net.
TUMOURS,
INNOCENT AND MALIGNANT:
THEIR CLINICAL CHARACTERS AND APPROPRIATE TREATMENT.
BY
J. BLAND-SUTTON, F.R.C.S.,
Surgeon to and Member of the Cancer Investigation Committee of the Middlesex Hospital, &c.
This work has been thoroughly revised by the Author, much new matter has been incorporated,
and many new illustrations have been added. The Author carefully discusses the Etiology of
Cancer in the light of the results of the most recent investigations.
JUST PUBLISHED.
TEXT-BOOK OF
GYNAECOLOGICAL SURGERY.
BY
COMYNS BERKELEY, M.A., M.D-, B.C.Cantab., F.R.C.P.Lond., Al.R.C.S.Eng.,
AND
VICTOR BONNEY, M.S., M.D., B.Sc.Lond., F.R.C.S.Eng., M.R.C.P.Lond.
With 16 Coloured Plates and 392 Illustrations in the Text. 720 pages demy 8vo. Cloth gilt, 25s. net.
DISEASES OF THE JOINTS AND SPINE.
By HOWARD MARSH, M.A., M.C.Cantab.,
F.R.C.S. New and Enlarged Edition,
, thoroughly Revised and partly Re-written
by the Author and by C. GORDON
WATSON, F.R.C.S. With many new Illus-
trations, including 11 Skiagrams and 4
Coloured Plates. 10s. 6d. net.
ELECTRICAL TREATMENT.
By WILFRED HARRIS, M.D.Cantab.,
F.R.C.P. Second Edition, Revised. Illus-
trated. 7s. 6d.
SERUMS, VACCINES, A^D TOXINES
IN TREATMENT AND DIAGNOSIS.
By W. C BOSANQUET, M.A., M.D.Oxon.,
F.R.C.P.Lond., and JOHN W. H. EYRE,
M.D., M.S.Dunelm, D.P.H.Cantab., F.R.S.
Edin. Second Edition, Revised. 7s. 6d.
SYPHILIS.
By Sir JONATHAN HUTCHINSON,
F.R.S., LL.D., F.R.C.S. New and Enlarged
Edition. With 36 new Plates, 12 in Colour
and 21 Black and White. 10th Thousand.
10s. 6d. net.
DISEASES OF THE SKIN.
An Outline of the Principles and Practice
of Dermatology.
By Sir MALCOLM MORRIS, K.C.V.O.
Revised Edition. With 10 Coloured and 47
Black-and-White Plates and other Illustra-
tions. litli Thousand. 10s. 6d.
RADIUMTHERAPY.
By Dr. LOUIS WICKHAM and Dr.
D6GRAIS. Translated by S. ERNEST
DORE, M.A., M.D.Cantab., M.R.C.P.
With a Preface by Sir MALCOLM
MORRIS, K.C.V.O., and Annotations by
Dr. WICKHAM. Illustrated. 15s. net.
CASSELL & CO., LTD., LA BELLE SAUVAGE, LONDON, E.C.
Gifts and Bequests
The Merchant Taylors' Company has given ?31 10s.
to the Samaritan Free Hospital.
Mrs. Isabella Tilson, of Stonehou^e, Gloucestershire,
has bequeathed ?50 to the Stroud Hospital.
The King has sent gifts of pheasants to the Mount
Yernon Hospital and the London Fever Hospital.
The Worshipful Company of Ironmongers has given
20 guineas to the Royal Hospital for Incurables, Putney
Heath.
The King has sent 20 guineas to the Royal National
Hospital for Consumption, Ventnor, and to the Maternity
Charity and District Nurses' Home, Plaistow.
The King has sent a gift of game for the uee of the
patients of the Royal Hospital for Incurables, Putney
Heath, of which institution His Majesty is Patron.
Miss Lucy Palmer-Morewood, of Leamington Spa,
Warwick, aged 93, has left ?100 to the Warneford Hospital,
Leamington, and ?50 to the Hospital for Incurables,
Leamington.
The Cloth workers' Company has sent 20 guineas as
an annual subscription, and the Cutlers' Company
10 guineas as a donation to the British Home and Hospital
for Incurables.
The Rev. Montague Earle Welby, M.A., of Terrace
Lodge, Richmond, Surrey, and lately rector of Uffington,
Stamford, Lines, who left estate valued at ?67,851 gross,
has left ?2,500 to the Middlesex Hospital, and ?2,500 to
the Lock Hospital and Rescue Home.
The following amounts have been received towards the
Prince Francis of Teck Memorial Fund of the Middlesex
Hospital: The Duke of Westminster, ?100; "Anony-
mous," ?26 5s.; "Anonymous" (per Mr. Charles Davis),
?25; Major A. G. C. Watson, ?20; Captain Bromley Way
(collection), ?13; Mr. W. M. Cazalet, ?10; Mr. S.
Neumann, ?105; Hospital Saturday Fund, ?50.
Mr. Jesse Adnitt has left ?50 to the Peterborough
Infirmary.
A eequest of between ?8,COO and ?10,000 has been left
to the Hastings, St. Leonards and East Sussex Hospital
by the late William Henin Christie.
Miss Finetta Sarah Hale, of Felixstowe, Suffolk, left
?100 each to the Suffolk General Hospital, Bury St.
Edmunds, and the Convalescent Plome at Felixstowe.
The Hon. W. F. D. Smith, chairman of the King's Col-
lege Hospital Removal Fund, for which a special appeal
is being made, has undertaken to pay the cost of one of
the central ward blocks of the new hospital. This will be
a three-storey block, and it is estimated that it will ccet
?30,000.
Among the latest contributions received at the Bank
of England for King Edward's Hospital Fund for Lon-
don are the following annual subscriptions : Mr. Walter
Morrison, ?5,000; Mr. William Waldorf Astor, ?1,000;
Messrs. C. J. Hambro and Son, ?250; Prudential Assur-
ance Company (Limited), ?250; Messrs. Wernher, Beit,
and Co., ?250; Mrs. Walter H. Burns, ?200.
Mr. Archibald Scott, lately a director of the London
and South Western Railway Company, who died on
December 6 last, aged ninety, left ?250 each to the Dundee
Infirmary, the Royal Hospital for Incurables, Putney, the
Surbiton Cottage Hospital, the Charing Cross Hospital, the
Westminster Hospital, and St. Thomas's Hospital.
Miss Anne Williamson, of Grove Street, Liverpool,
who died on December 23, aged ninety-one, daughter of the
late Mr. John Williamson, left the ultimate residue of her
estate, subject to a life interest, as to two-sixths to the
Orphan Asylum, Myrtle Street, Liverpool; and as to one-
sixth each to the Liverpool Bluecoat School, the Royal
Infirmary, Liverpool, the Children's Infirmary, Liverpool,
Seamen's Orphan Arylum, Liverpool.
Some little confusion has recently been caused by circu-
lars requesting biographies for a publication entitled " The-
International Who's Who." This nuisance is not likely to-
recur, as Mr. Justice Parker in the High Court of Justice
has, on the application of the proprietors of " Who's Who,"
Messrs. A. and C. Black, in addition to other relief.,
granted an injunction restraining the proprietors of " The'
International Who's Who " from publishing that book iiv
the form previously produced which infringed the copy-
right in "Who's Who," and further restrained them from
using the title of " The International Who's Who " in any
publication without clearly distinguishing it from Messrs.
A. and C. Black's publication.
At the Times' Book Club in Oxford Street a sale of
second-hand books is in progress. To medical men
the chief interest will be the corner of the shop devoted
to medical and surgical literature, where a large number
of volumes on every department of professional activity
are on show. The critical purchaser will find that the
prices set upon the books vary considerably, and have pro-
bably been fixed without much consultation with a medical
assessor. There are some quite recent editions of standard
authorities priced most extraordinarily cheap?some only
a fifth or a sixth of the published price; while there are
others which are hopelessly out of date, but are marked
down only 50 per cent, or so. Again, on one shelf are
to be seen two copies of the same edition of a well-known
textbook on diseases of the eye; the cleaner of the two is
ticketed 6s., the other 9s. 6d. Still, these in-
stances also Ghow that the discriminating purchaser who
is content to wander from one shelf to another can get some
notable bargains. At the same time it is safe to say that
the volumes most worth acquisition at the prices asked will
speedily disappear in a shop so near Cavendish Square, and
those who like to replenish their libraries without great
expense may be recommended to visit the Times' Book
Club forthwith.
BUILDINGS CONTEMPLATED AND
IN PROGRESS.
Ayr.?Under the superintendence of the Architect,
Mr. J. K. Hunter, an important work in the installation
of a new heating system has been carried out at the Ayr
County Hospital. The work has ccst about ?2,000.
Edinburgh.?Mr. A. F. Balfour Paul is architect for
the important extension to the Hospital for Women and
Children; it is a contributory establishment, and offers to
all the advice of medical experts of their own sex. The
addition comprises fourteen new beds and the necessary
auxiliaries, dispensary, improved administration offices, and
a new operating theatre with anaesthetic and sterilising
rooms. The whole accommodation now gives forty beds for
patients and cubicles for nurses, with laundry, wash-house,
testing laboratory, etc., in a separate outbuilding. The
new work is built of stone, of fire-resisting construction
throughout, and heated on the low-pressure hot-water
system.
Glasgow.?The ophthalmic hospital and dispensary are
to be reconstructed.
Goole.?A new cottage hospital is to be built at Goole,
to replace the present institution, which has served for
twenty j-ears.
Liverpool.?The select vestry have decided to erect an
epileptic colony at Maghull at an estimated cost of
?39,030. A tender of over ten thousand pounds has been
accepted for the proposed alterations to Brownlow Hill
Workhouse.
Norwich.?Mr. E. T. Boardman is the architect for the
new Norfolk and Norwich Hospital, of which the late
King laid the foundation stone in the latter part of 19C9.
The isolation block was formally opened last week, and
the contract sum was ?5;856.
Southport:?Mr. F. P. Halsall, A.R.I.B.A., is architect
for the Infirmary extension, costing ?5,000. It will com-
prise four small wards, containing eleven beds, and a re-
modelling for extended administrative accommodation.
Stoke-on-Trent.?Thirty thousand pounds is the sum
required for the remodelling of the North Staffordshire
Infirmary according to the report of the structural sub-
committee assisted by the architects, Messrs. Scriven?r
and Sons.
Swansea and Merthyr Tydfil.?The visiting com-
mittee are now advertising for an architect for the erection
of a joint asylum. Applications to be sent to the clerk
before February 15.
Ulster.?Messrs. Watt, Tulloch, and Fitzsimons, of
Belfast, are architects for the Ulster Hospital for Chil-
dren and Women in Templemore Avenue, Belfast, for
which estimates are now being invited.
February 18, 1911. THE HOSPITAL
621
Jottings.
The annual meeting of the governors of the Royal
National Orthopaedic Hospital will be held at 234, Great
Portland Street, W., on Wednesday, March 1.
In memory of King Edward VII. a bed has been endowed
at Charing Cross Hospital to be called for ever " The King
Edward VII. Bed," and Princess Christian has been asked
to unveil the tablet erected in the Queen Victoria Ward.
The Annual Court of King's College Hospital will be
neld in the Board Room, on Thursday, February 23, at
five o'clock, when will be presented a report of the receipts
and disbursements of the past year and of the general state
of the hospital, and other special business will be trans-
acted.
The King's Letter to the Nation, the entire proceeds of !
the sale of which will also be devoted to some charitable (
institution to be selected by his Majesty, will be issued to j
the public about a month before the Coronation. The !
allegorical border to the King's Letter has been designed |
by Sir Lawrence Alma-Tadema, 11.A.
Efficient means of fire protection at asylums and institu- i
tion.s is an undoubted necessity. The Stirling District Board |
of Lunacy has recently decided on improving the existing
fire extinguishing arrangements at the asylum at Larbert,
with the result that a powerful pump of the Merryweather
*' Midland " type has been installed for the purpose of
augmenting the water-pressure, which was insufficient to
secure a satisfactory jet for fire extinction. The new
pump will enable a powerful jet to be brought to bear at
any point where a fire may occur.
The Lord Lieutenant of Ireland and the Countess of
Aberdeen, the Lord Chancellor of Ireland and Lady
Walker, and the Lord Mayor and the Lady Mayoress
of Dublin have given their patronage to a ball which
will be held at the Gresham Hotel, Dublin, on February 22,
22 in aid of the Jervis Street Hospital.
It is not surprising to learn that the British Home
and Hospital for Incurables has gained much support in
response to the appeal which it issued last December.
It was a wise step to employ Mr. George R. Sims as pro-
tagonist on behalf of the Home, and his pamphlet " The
Cry of the Helpless " is as readable as a facile journalist
can make it. The secretary of the Home is to be con-
gratulated on the postcard which accompanied the book
with its request for the address of a friend to whom the
first reader might have a copy of the appeal sent. The
middle class, for whom the Home was founded, could
have found no better advocate than Mr. Sims.
The Duke of Rutland presided over a representative
meeting held last week, when an Executive Com-
mittee, which included the Lord Lieutenant, the High
Sheriff, the Chairman of the County Council, the Mayors of
Leicester and Loughborough, Sir Charles McLaren, K.C.,
M.P., Sir Arthur Hazlerigg, Bart., Sir Edward Wood,
J.P., Mr. G. Murray Smith, D.L., J.P., the Recorder of
Leicester, Mr. T. Fielding Johnson, J.P., Mr. F. Hewitt,
and Mr. A. Banton, were appointed to consider sugges-
tionSi for the proposed Leicestershire Memorial to King
Edward VII., one of which is the endowment of beds at the
Leicester Infirmary.
TO HOSPITAL SECRETARIES AND OTHERS.
W E invite contributions from any of our readers, and
shall be glad to pay, according to length, for items of news
for the institutional section (including appointments, resig-
nations, gifts, etc.) which we may publish. All payments
for manuscripts used are made as early as possible after
the beginning of each quarter.
JVTEDICAL APPOINTMENTS.
Full particulars of these vacancies can be obtained on
reference to our advertisement columns :?
Evelina Hospital.?House Physician.
Leeds Public Dispensary.?Junior Resident Medical
Officer.
Manchester Children's Hospital.?Resident Medical
Officer.
Manchester Royal Infirmary.?House Physicians and
House Surgeons.
Royal Infirmary, Manchester.?House Physicians and
House Surgeons.
Tower Hamlets Dispensary.?Resident Medical Officer.
West Bromwich District Hospital.?Assistant Resident
House Surgeon.
Un*d::r a new editor, who is evidently determined to
make his term of office memorable, the St. Thomas Hospital
'Gazette, has appeared in a new and most artistic cover, far
superior to anything of the kind prevailing among the
other medical school organs. Nor do the contents of the
first number belie the promise of this pleasing exterior.
It is with great .pleasure that we offer congratulations to
the editor on his energy and keenness, and beet wishes for
a large circulation as a result of his venture.
THE WEEK'S NOVELTIES.
The ROYAL DOULTON POTTERIES COMPANY,
Albert Embankment, has been entrusted with the entile
installation cf sanitary appliances for the public conveni-
ences at the approaching Sccttkh Historical Exhibition,
Glasgow. A thoroughly representative exhibit of the
various manufactures of the Company will be made at the
same Exhibition.
Every clay the knowledge of the dangers and expensive-
ne/5S'of dust brings new recruits to the only sensible, practi-
cal, easy and perfect way of keeping it in check, by means
of a DAISY VACUUM CLEANER. Dust cannot be satis-
factorily dealt with save by the vacuum system; it is not
enough to chase it from floor to furniture, from furniture
to shelves and pictures and back again with broom and
duster ; it were wiser to allow it to rest undisturbed than to
send it into the atmosphere with all its attendant risks
from germs of all kinds, for in dust of every description
the germ finds a most .suitable resting place. Everyone
who realises that dust ha.3 no place in the really clean and
therefore healthy institution mutt of necessity relegate the
broom, brush, duster and dust pan to the past; they are
not only unsatisfactory in operation, but they are creators
of work in an age when everything that can be done to
minimise labour is being done. With a Daisy Vacuum
Cleaner all duet, not a portion merely, can be captured
from furniture and fittings, and carried in its air-tight
cage out of the house away to a safe distance, where it
becomes no longer a source of worry. rick, and work. The
Daisy is offered at prices which will bring about its u.30
in cottage, villa, mansion, hotel, public institutions, and
palace alike. A copy of an illustrated book which gives
prices and particulars of all the models may be had for the
asking from the Daisy Vacuum Cleaner Co., Ltd.,
Leamington Road, Gravelly Hill, Birmingham.
"The Uses and Modes of Application of Adhesive
Plasters."?A translation from that standard work on
bandaging, Hofmeister's " Verbandtechnik," is a neat
booklet which is sent out by Beiersdorf & Co., of
Hamburg who are the makers of the now so popular
" LEUKOPLAST." We have already stated that we
consider Leukoplast one of the best of adhesive piasters
on the market, and this little exposition of its manifold
uses and possibilities will be of service to those who already
know of it, while it will be an eye-opener to those who are
not yet acquainted with its excellences. We advise every
practitioner who does not keep a supply of Leukoplast in
his surgery to write to the London olliee, 7 and 8 Idol Lane,
E.C., for a sample and a copy of this booklet.
The question of PURIFYING WATER BY MEANS
OF OZONE has been earnestly studied by Ozonair, Ltd..
a firm which is the sole manufacturer of ozone purifying
apparatus in this country. The Company has already
several large orders in hand for installations and the im-
portance of the matter, to institutional workers especially,
can scarcely be overrated. The cost of purifying water by
this new system is below Id. per 1,0C0 gallons, and
the apparatus is simple and thoroughly efficient. The
Company supplies an illustrated booklet on the subject,
which may be obtained on application to headquarters,
Ozonair, Ltd., 9G Victoria Street, S.W.
The Times' Bock Club announces a special sale of
MEDIC vL BOOKS at startling reductions. The volumes
are excellent, clean copies of standard works, and are well
worth the attention of practitioners. Particulars of the
sale appear in our advertisement columns.
BUSINESS NOTICES.
Annual Subscriptions.
The rate of annual subscriptions to The
Hospital, either direct from the Office or through
agents, is: ?
For the United Kingdom  15s. a year.
For the Colonies and Abroad ... 17s. ,,
For the United Kingdom (with
Insurance Policy)  ?1 ,,
inclusive of postage in each case.
Letters relating to the Publishing, Sale and
Advertisement Departments must be addressed to
-the Manager (not to the Editor): ?
The Manager,
The Hospital Building,
28 and 29 Southampton Street,
Strand, London, W.C.
The Relaxations of Medical Men.
We shall also be glad to pay for accepted contri-
butions, from any member of the profession, on the
subject of the relaxations of practitioners. This
opens up a wide field, as it includes natural history,
photography, sport, indoor recreations, and motor-
ing. Whenever possible, original illustrations and
photographs should be sent with the MS.
Correspondence.
Correspondence on all subjects is invited, but no
communication can be entertained if the name and
address of the correspondent are not given as a
guarantee of good faith, but not necessarily for pub-
lication. All correspondents should write on one
side of the paper only.
February 18, 1911 THE HOSPITAL
The Scientific Press Publications
INSTITUTIONAL WORKS.
HOSPITALS AND ASYLUMS OF THE WORLD.
By SIR HENRY BURBETT, K.C.B., K.C.Y.O.
In Four Volumes1' with Separate Portfolio^containing severa'^hundred Plans.
Vols. I. and'II. (ASYLUMS)] ...SPriee ?6 15s. I Vols. III. and IV. (HOSPITALS)
and the Portfolio of Plans ... Price ?9
The Portfolio of Plans (20 in. x 14 in., drawn to unifornr'scale), Price :?4 14^,6
ThelComplete Work   ?12 12s.
This magnificent work forms a Complete Survey of the Origin, History, Construction, Administration and Management
of the Hospitals and Asylums of the World, with detailed information concerning the principal Institutions.
Only a few copies remain unsold.
LEDGERS, ACCOUNT BOOKS, STOCK BOOKS of all kinds for large
and small Institutions.
These are supplied already ruled and printed, or in accordance with the special requirements of institution managers.
ANNUAL REPORTS AND PROSPECTUSES.
The Scientific Press are prepared to undertake the production of the Annual Reports and Prospectuses of every
class of Institution, and to supply printed or typewritten copies of testimonials for the officials. Professional Cards.
THE SCIENTIFIC PRESS, LTD.,
28 and 29 Southampton Street, Strand, London, W.C.

				

